# Understanding Why Lateral Osteotomy During Rhinoplasty Can Be Performed Safely

**Published:** 2019-04-01

**Authors:** Arian Mowlavi, Jay B. Kim, Natalia Molinatti, Sean Saadat, Soheil Sharifi-Amina, Bradon J. Wilhelmi

**Affiliations:** ^a^Cosmetic Plastic Surgery Institute, Laguna Beach, Calif; ^b^University of California, Irvine; ^c^Washington University in St Louis, St Louis, Mo; ^d^George Washington University School of Medicine & Health Sciences, Washington, DC; ^e^Riverside Community Hospital, Riverside, Calif; ^f^Hiram C. Polk Jr Department of Surgery, University of Louisville, Louisville, Ky

**Keywords:** osteotomy, rhinoplasty, nasal bridge, safety, nose job

## Abstract

**Background:** Lateral osteotomy is a mainstay of rhinoplasty surgery and involves fracture of the nasal and maxillary bones to narrow or widen the nasal dorsal bridge and base. To avoid nasal midvault collapse following rhinoplasty, the accepted “high-low-high” lateral osteotomy technique advocates for the preservation of a triangular strut of maxillary bone when initiating the osteotomy. **Objective:** We evaluated the risk of starting a lateral osteotomy in the “high” position to leave the aforementioned triangular maxillary strut without risk of falling into the nasomaxillary suture line, which can result in an aberrant and uncontrolled fracture. **Methods:** We utilized high-definition computed tomographic scans to reconstruct layered 3-dimensional images of 20 patient skulls and measured the distance from the rhinion (most inferior point of the central nasal bone junction) to the nasomaxillary suture line and from the rhinion to the maxillary groove. **Results:** We found that the nasomaxillary suture line was reliably only halfway down the bony nasal pyramid and not in proximity to the maxillary groove. **Conclusions:** Our findings provide reassurance that a generous triangular strut can be preserved along the maxillary component of the piriform aperture without concern of falling into the nasomaxillary suture line. Thus, controlled lateral osteotomies can be performed safely to achieve aesthetic gains without fear of compromising midvault stability.

Plastic surgeons consider rhinoplasty to be one of the most challenging facial procedures. Because of its central location, the nose plays a critical role in facial aesthetics, rendering any alteration to its appearance vulnerable to critique. Moreover, optimal rhinoplasty results require an improvement in the appearance of the nose without harming the delicate functional aspect of the nasal airways; thus, a surgeon performing a rhinoplasty must consider nasal function while addressing any aesthetic concerns.

The bony support structure of the nose is composed of a pair of nasal bones that attach superiorly to the frontal bone and laterally to the frontal process of the maxillary bones. Together, this ascending portion of the maxilla and the nasal bones form the bony nasal vault, which forms the upper nasal third and lends support to the cartilaginous structures that comprise the rest of the nose. Mobilizing the bony support structure of the nose via osteotomy is a standard component of rhinoplasty surgery—of particular interest is the lateral osteotomy, commonly used to rectify deformities of the nasal pyramid by allowing a surgeon to change its width and/or projection.^1^

The most widely accepted technique for the lateral osteotomy nowadays was popularized by Webster et al^2^ in 1970.^1^^,^^3^ This technique, termed the “high-low-high” lateral osteotomy, involves a curved cut that begins “high” on the piriform aperture at the level of the attachment of the inferior turbinate. The cut turns and proceeds upward deep in the maxillary groove (the “low” portion) before curving superiorly and anteriorly, terminating inferiorly to the nasofrontal suture line (thus termed “high”). Starting high on the piriform aperture allows for the preservation of a small triangle of maxillary bone extending medially and anteriorly, termed “Webster's triangle,” that supports the lateral nasal cartilages. Lateral osteotomy techniques prior to this, including the “low-to-low” lateral osteotomy, were effective in bringing about aesthetic improvement but were wrought with postoperative complications, such as midvault collapse and the obstruction of the nasal airway.^2^^,^^3^^,^^4^

Despite this well-regarded “high-low-high” technique, performing a predictable and controlled osteotomy remains a concern to less experienced rhinoplasty surgeons. Complications can arise when the bones fracture in an unwanted direction, producing, at minimum, a suboptimal aesthetic result.^5^^,^^6^ To mitigate the likelihood of an uncontrolled fracture, avoiding a cut in close proximity and near parallel to the nasomaxillary suture line is a crucial aspect of optimal osteotomy technique. A lateral osteotomy too close to this suture line increases the risk of an uncontrolled fracture, as the nasal and maxillary bones increase in thickness near the suture lines.^6^ These areas will require the greatest osteotome forces to cut and hence involve the greatest risk of uncontrolled fracture when these forces are transmitted to thinner areas of bone.^6^ In this radiologic study, we take a closer look at the anatomic bony structures relating to the “high” start of the lateral osteotomy and evaluate the reliability of this technique against the risk of a complication caused by encroaching upon the nasomaxillary suture.

## METHODS

**Figure 1:**
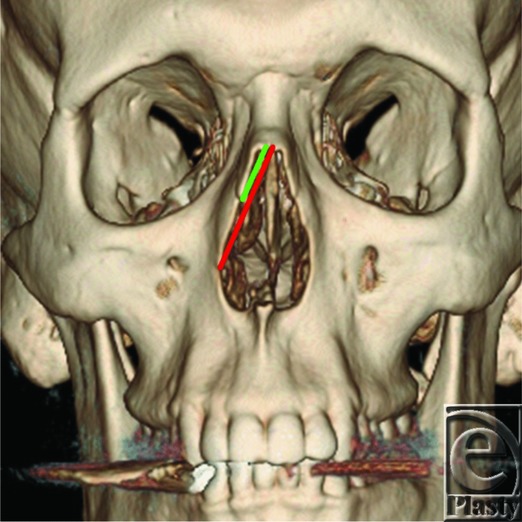
A 3-dimensional reconstruction image measured in this study. Green line illustrates measurement from rhinion to nasomaxillary suture. Red line illustrates measurement from rhinion to maxillary groove.

This study's protocol was reviewed and confirmed as nonhuman subject research by the St Joseph Health Center for Clinical Research (Irvine, Calif). Thus, institutional review board approval was not required. Computed tomographic (CT) scans of 20 patients, 10 males and 10 females, with an average age of 37.2 ± 19.1 years, were examined in this study. Patients who had incurred damage or surgical modification (rhinoplasty) to the bony nasal vault were excluded. We created high-resolution 3-dimensional (3D) reconstructions of patient skulls using a cross-sectional slice thickness of 0.6 mm. Of each CT scan, 2 straight measurements were taken for each side of every nasal pyramid, along the outer edge of the piriform aperture: (1) the distance from the rhinion to the nasomaxillary suture, and (2) the distance between the rhinion and the maxillary groove (Fig 1). The measurements made to the inferior part of the maxillary groove were terminated at the deepest and most well-defined point of this landmark in each image. Measurements were taken using the anteroposterior view of each skull. The ratios between the distances were calculated and expressed as a mean ± standard deviation. The data were analyzed for statistical significance using Student's *t* test.

## RESULTS

The average distance from the nasal midline to the nasomaxillary suture was 9.30 ± 1.47 mm, and the average distance from the nasal midline to the maxillary depression was 18.5 ± 2.35 mm. Our data demonstrate a statistically significant difference between the position of the nasomaxillary suture line and the maxillary groove (*P* = 8.58 × 10^−35^). The mean ratio between these 2 measurements was 0.501 ± 0.0415. There was no significant difference between corresponding left and right distances (*P* = .488) and no gender difference between the mean ratios (*P* = .631). Our results suggest that the nasomaxillary suture line is, on average, only halfway down the nasal pyramid and not in proximity to the maxillary groove.

## DISCUSSION

The lateral nasal osteotomy is indispensable to a surgeon looking to optimize rhinoplasty results. By nature, osteotomy is a technique prone to inconsistency, as bones are at risk of fracturing uncontrollably along points of weakness and/or if too much force is applied.^5^^-^7 Thus, the ideal technique for the lateral osteotomy should be consistently reproducible, while being safe and sufficient to achieve the aesthetic and functional goals of the encompassing rhinoplasty.^8^

The “high-low-high” lateral osteotomy technique most commonly used nowadays reduces the risk of postoperative nasal midvault collapse by virtue of “Webster's triangle,” the triangular strut of maxillary bone left intact when the surgeon begins the osteotomy high on the piriform aperture.^2^^,^^3^^,^^4^ However, this raises concerns over whether a surgeon risks an uncontrolled fracture by starting the cut too high, near the suture where the nasal bone meets the ascending process of the maxilla.^6^ Using CT reconstructions of patient skulls, our study took a closer look at the anatomy of the bony nasal vault to evaluate this hypothetical risk.

Our data indicate that the nasomaxillary suture line is not in proximity to the maxillary groove. In fact, the distance from the nasal midline to the nasomaxillary suture was, on average, remarkably close to one-half of the distance from the nasal midline to the maxillary depression. Our findings provide reassurance that the “high-low-high” lateral osteotomy can be performed safely because a generous triangular strut can be preserved on the piriform aperture without the risk of starting the cut within the nasal bones or too close to the nasomaxillary suture line. In addition, because this suture line is only about halfway down the nasal pyramid and not in close proximity to the maxillary depression, a surgeon can complete the “low” portion of the lateral osteotomy up the thinner bone of the maxillary groove^6^ with reassurance that the cut path will not fall too close to the nasomaxillary suture. The cut can then be curved medially, terminating in the “high” position inferiorly to the nasofrontal suture. This is critical not only to prevent a rocker deformity^1^^,^^9^ but also because it allows the surgeon to cut across the nasomaxillary suture before the bones become too thick; this affords the surgeon greater control over the osteotomy and lowers the risk of an uncontrolled fracture by minimizing the required osteotome forces.^6^ Our findings are applicable regardless of whether the osteotomy technique is internal or external.

**Figure 2:**
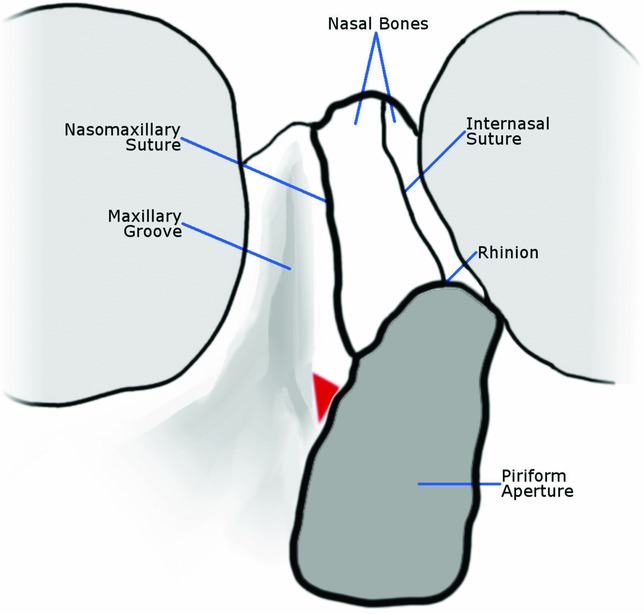
A typical illustration of nasal bony anatomy. Webster's triangle is highlighted in red. Note how nasomaxillary suture appears to be in close proximity to the maxillary groove.

It is easy to overestimate the contribution of the nasal bones to the projection of the nasal pyramid. Thus, it may not be apparent to the young surgeon that most of the lateral osteotomy takes place in the maxillary bone. We conducted this study partly because we noticed that illustrations of the bony nasal vault tend to exaggerate the size of the nasal bones in relation to the nasal process of the maxillary bones; these illustrations often misrepresent the position of the nasomaxillary suture line very low on the bony nasal vault (Fig 2). In contrast, our findings provide evidence that the maxillary bones comprise the entire bottom half of the nasal pyramid.

The “high-low-high” method remains an effective technique for lateral osteotomies, allowing a surgeon to change the width of the nasal base, straighten a deviated nose, or close an open roof after hump reduction, while minimizing the likelihood of postoperative deformities or nasal airway obstruction.^4^^,^^7^^,^^10^ We hope that this study will provide further knowledge to practitioners of rhinoplasty surgery and reinforce their ability to perform “high-low-high” lateral osteotomies safely and effectively.
